# Protection against homo and hetero-subtypic inﬂuenza A virus by optimized M2e DNA vaccine

**DOI:** 10.1080/22221751.2018.1558962

**Published:** 2019-01-16

**Authors:** Yanfeng Yao, Huadong Wang, Jianjun Chen, Zhiyong Shao, Bin He, Jie Chen, Jiaming Lan, Quanjiao Chen, Ze Chen

**Affiliations:** a National Biosafety Laboratory, Wuhan Institute of Virology, Chinese Academy of Sciences, Wuhan, People’s Republic of China; b Institute of Animal Husbandry and Veterinary Science, Wuhan Academy of Agricultural Science and Technology, Wuhan, People’s Republic of China; c Center for Brain Science, Key Laboratory of Magnetic Resonance in Biological Systems and State Key Laboratory of Magnetic Resonance and Atomic and Molecular Physics, Wuhan Institute of Physics and Mathematics, Chinese Academy of Sciences, Wuhan, People’s Republic of China; d CAS Key Laboratory of Special Pathogens and Biosafety, Chinese Academy of Sciences, Hubei, People’s Republic of China; e Department of Pathogenic Biology, Hebei Medical University, Shijiazhuang, People’s Republic of China; f College of Life Science, Hunan Normal University, Changsha, People’s Republic of China; g Shanghai Institute of Biological Products, Shanghai, People’s Republic of China

**Keywords:** Influenza, M2e, tPA, optimized M2e DNA vaccine, Universal influenza vaccine

## Abstract

Current influenza vaccines provide hemagglutinin strain-specific protection, but rarely provide cross-protection against divergent strains. It is, therefore, particularly important to develop a universal vaccine against conserved proteins or conserved regions of the virus. In this study, we used N-terminal extracellular region of the influenza virus M2 protein (M2e) as the target antigen and constructed two optimized M2e DNA vaccines (p-tPA-p3M2e and p-p3M2e) with increased antigenic epitope density and enhanced antigen secretion. Both vaccines induced high M2e-specific humoral and cellular immune responses in the vaccinated mice. These two vaccines also conferred protection against a lethal infection of homo-subtypic H1N1 virus, with p-tPA-p3M2e being the most effective. In addition, p-tPA-p3M2e also showed cross-protection against different subtypes of the influenza virus (H9N2, H6N6, and H10N8) at varying rates (80%, 40%, and 20%, respectively). After passive immunization, M2e DNA vaccine-induced antibodies in the sera provided complete protection against homologous virus challenge. An analysis of the mechanism underlying this immunization-mediated protection indicates that M2e-specific IgG and T-cell immune responses may play critical roles in the prevention of infection and viral clearance. Taken together, our results indicate that this optimized M2e DNA vaccine is a promising candidate for the development of a universal, broad-spectrum influenza virus vaccine.

## Introduction

Influenza is caused by the influenza virus, an important human respiratory infectious disease, which causes 250,000–500,000 deaths worldwide every year [1]. In recent years, drug-resistant strains and new types of influenza are continuously emerging, causing the influenza epidemic to be a constant and serious issue [2,3]. Vaccinations are the most effective way to control influenza. However, most of the influenza vaccines currently available are subtype-specific and fail to protect patients against other types or antigenic variants of the virus. Furthermore, the influenza virus undergoes frequent and unpredictable mutations that necessitate annual vaccine updates [4,5]. This not only limits the applicability of the vaccine but also wastes manpower and material resources. Thus, the development of universal vaccines that provide broad protection to control seasonal influenza epidemics and the occasional outbreak of pandemics is essential.

Influenza virus M2 protein is an integral membrane protein expressed on the viral surface in low quantities, while being abundantly present on the surface of infected cells [6,7]. This expression pattern plays an important role in viral replication. The M2 protein consists of an N-terminal extracellular region (M2e), transmembrane region, and C-terminal cytoplasmic tail region. The M2e is composed of 24 amino acids and is highly conserved among different influenza A subtypes [8]. Thus, this region may be a good candidate epitope for the preparation of a universal vaccine. However, in its natural state, viral M2e has low immunogenicity and abundance. To improve immunogenicity, previous studies have used multimeric forms of M2e created by fusing M2e to highly immunogenic carriers or by applying them in conjunction with adjuvants. The main types of vaccines include peptide vaccines, recombinant protein vaccines, and virus-like particles [9–13]. Currently, several M2e universal vaccines have entered clinical trials. However, some problems surfaced during clinical trials, which prevented the commercialization of these vaccines [13,14]. Some of the more prominent problems include unsatisfactory immune efficacy and certain side effects. These problems may be due to the vaccine containing multiple ingredients [15]. Moreover, this type of vaccine has a relatively high cost of production and a complex manufacturing technique, which further limits its development. Therefore, further research on M2e vaccine is necessary. Among the various types of vaccines, DNA vaccines are particularly attractive because of their simplicity, ease of production, and lack of anti-vector immune responses. Though the development of novel cross-protective influenza vaccines based on M2e DNA is a promising strategy, few of the enhanced immunogenic vaccine strategies have been successful. Thus, further research is necessary.

In this study, we used a combination strategy to improve the immunogenicity of the M2e DNA vaccine. First, we increased M2e epitope density using a combined gene construct containing three M2e genes in tandem (3M2e). Next, 3M2e gene codon optimization (p3M2e) was performed to increase expression. Finally, we coupled p3M2e with the secretory signal sequence of tissue plasminogen activator (tPA) to form tPA-p3M2e to improve protein secretion. These enhanced M2e DNA vaccines were then tested in mice to determine the protective effects against the influenza virus. To our knowledge, this is the first time an optimized M2e DNA vaccine with these particular modifications has been tested *in vivo* against both homologous and heterologous viruses.

## Results

### Enhanced expression and secretion of optimized M2e in vitro

**Figure 1.**
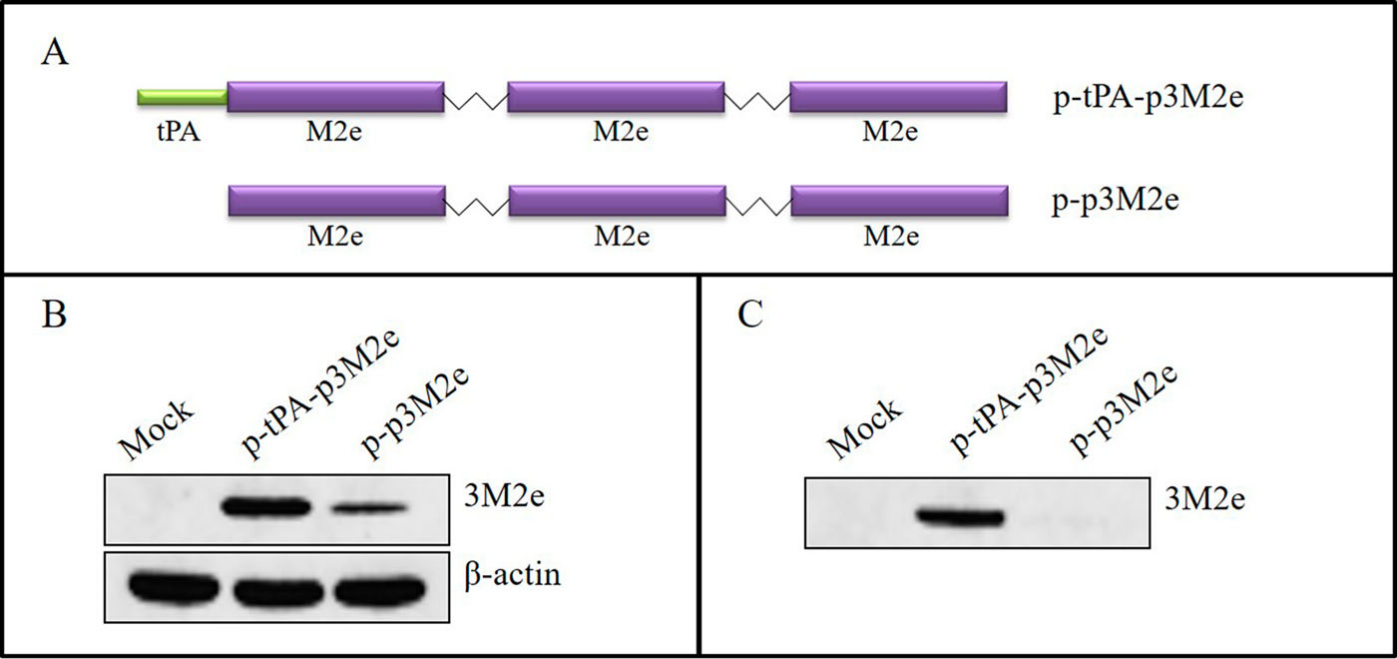
Construction and characterization of the M2e DNA constructs. (A) Schematic diagram of different forms of M2e in the pVAX1 vector. Three tandem copies of M2e were conjugated with linkers (p3M2e), and tPA was fused to the N-terminus (tPA-p3M2e). (B) Expression of different forms of M2e in 293T-cell lysates. (C) Expression of different forms of M2e in 293T-cell supernatants.

Three sequential repeats of the M2e gene, conjugated with linkers, were joined to the C-terminus of tPA (p-tPA-p3M2e) or without tPA (p-p3M2e) (Figure 1(A)). To detect expression *in vitro*, p-p3M2e and p-tPA-p3M2e were transfected into 293T cells and western blotting was performed. As shown in Figure 1(B) and (C), the p-tPA-p3M2e was more highly expressed than p-p3M2e in the cell lysates and supernatants. In fact, M2e protein expression was undetectable in the supernatants of the cells transfected with p-p3M2e. These data indicate that the addition of the tPA signalling sequence greatly enhanced the expression and secretion of 3M2e protein in 293T cells.

### Enhanced humoral and cellular immune effects are observed following optimized M2e DNA construct immunization in mice

**Figure 2.**
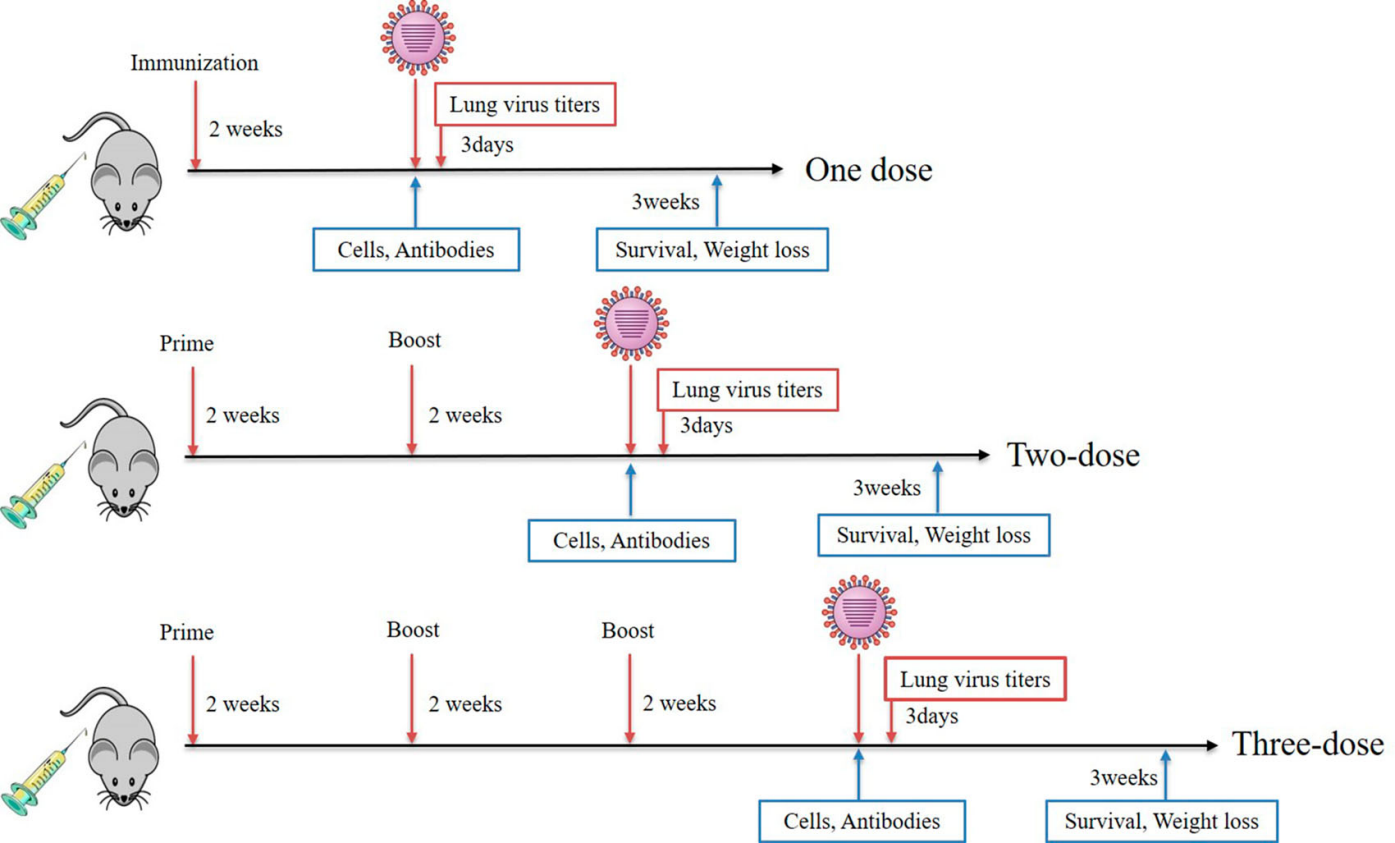
Schematic of the study. BALB/c mice were immunized once, twice or three times with DNA vaccine, however, the control groups were immunized three times with control plasmids. Sera were collected at the indicated times to analyse the humoral immune response and spleen cells were isolated for ELISpot assay. In parallel experiments, the remaining mice were challenged with a lethal dose of mouse-adapted viruses to detect the protective effect.

**Figure 3.**
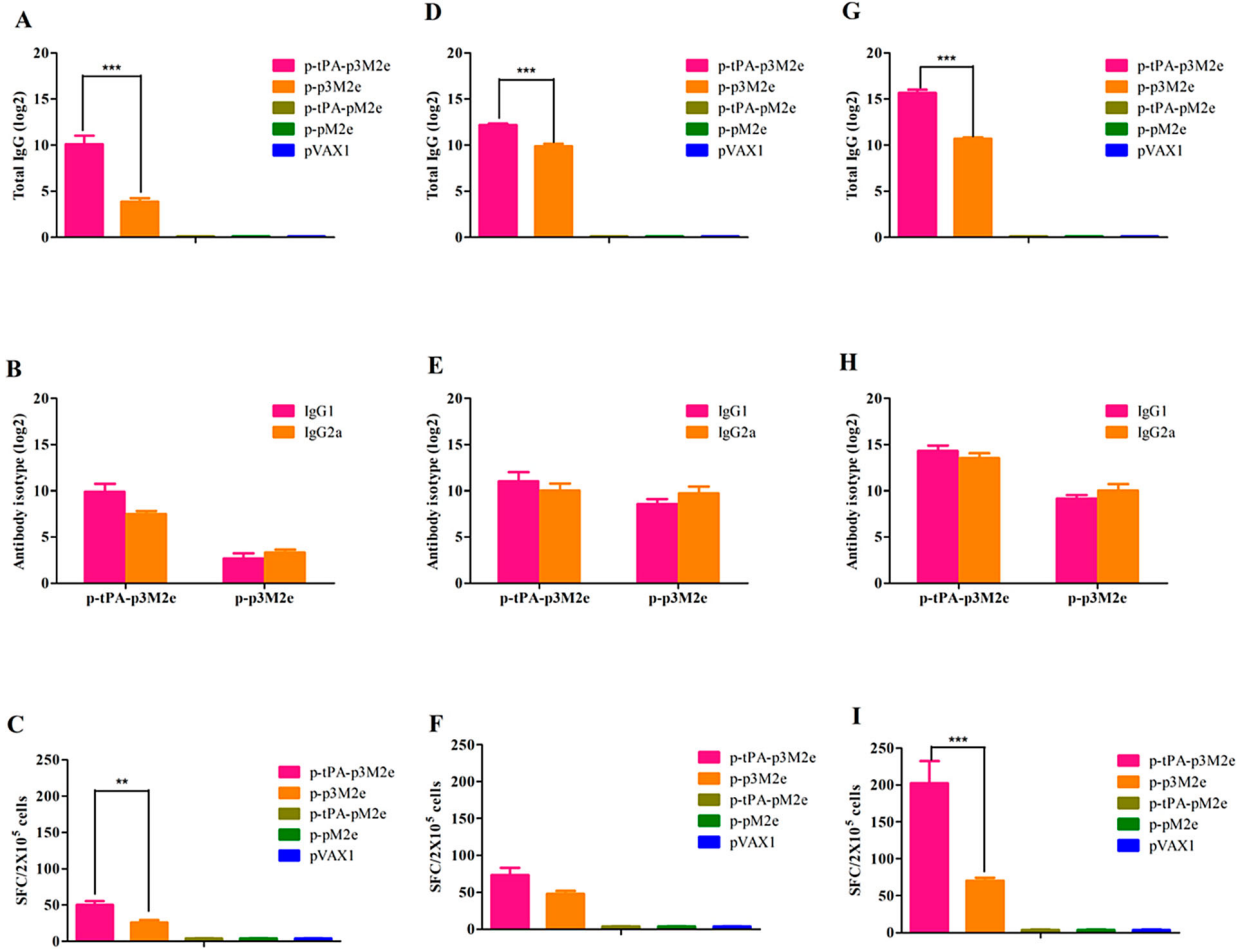
M2e-specific humoral and cellular immune responses. M2e-specific IgG, IgG1, and IgG2a titers were determined by ELISA. Cellular immune response induced by M2e-immunized mice detected by ELISpot. Data are expressed as spot-forming cells (SFCs) responding to peptide-specific IFN-γ secretion and are presented as the means ± standard deviation. [A, B, C (once immunization), D, E, F (twice immunization) and G, H, I (three times immunization)]. Statistical significance was defined as ***p < 0.001, **p < 0.01.

BALB/c mice were vaccinated with 50 μg of the different DNA constructs. Figure 2 shows the scheme followed for these experiments. The levels of M2e-speciﬁc antibodies were determined with enzyme-linked immunosorbent assay (ELISA) using synthesized M2e peptide as the antigen. Our results show that M2e-specific antibodies are present in both the p-tPA-p3M2e and p-p3M2e immunized groups 2 weeks after the initial immunization (Figure 3(A)). Furthermore, while antibody titer increased following each immunization for both groups, the levels were higher in the group immunized with p-tPA-p3M2e than those observed for the p-p3M2e group (Figure 3(A), (D), (G)). Notably, no M2e-specific antibodies were detected in the p-tPA-pM2e, p-pM2e, or pVAX1 immunized groups. To further evaluate the T-cell-related immune effects of p-tPA-p3M2e and p-p3M2e, specifically the Th1/Th2 immune response, we tested the IgG1 and IgG2a titers in the serum samples. High levels of IgG1 and IgG2a antibodies were induced in both the p-tPA-p3M2e and p-p3M2e immunized groups, suggesting that they both induce a Th1/Th2-related immune response (Figure 3(B), (E), (H)). Moreover, the M2e-specific IgG1 and M2e-specific IgG2a titers were similar, indicating that p-tPA-p3M2e and p-p3M2e induced a balanced Th1/Th2 immune response. To determine whether the antibodies produced following M2e DNA vaccination recognize native M2 protein, we performed a whole-cell ELISA assay. Our results show that the serum of mice immunized with p-tPA-p3M2e and p-p3M2e specifically binds to H1N1-infected MDCK cells, indicating that the M2e-specific antibodies induced by p-tPA-p3M2e and p-p3M2e immunization recognize the M2 protein on the surface of cells infected with influenza virus (Figure S1).

Secretion of IFN-γ by spleen cells is known to reflect the cellular immune response after immunization. As shown in Figure 3(C), the number of spots produced after two immunizations with p-tPA-p3M2e and p-p3M2e was significantly higher than that of the control group (*p *< 0.01). The number of spots also gradually increased with each immunization for both groups (Figure 3(F), (I)). These results further confirm the induction of an M2e-specific Th1/Th2 cell response following immunization with p-tPA-p3M2e and p-p3M2e. The control group also had several nonspecific spots (spot number ≤10/10^6^ cells).

### DNA vaccination with optimized M2e confers enhanced protection against homologous influenza virus challenge in mice

**Figure 4.**
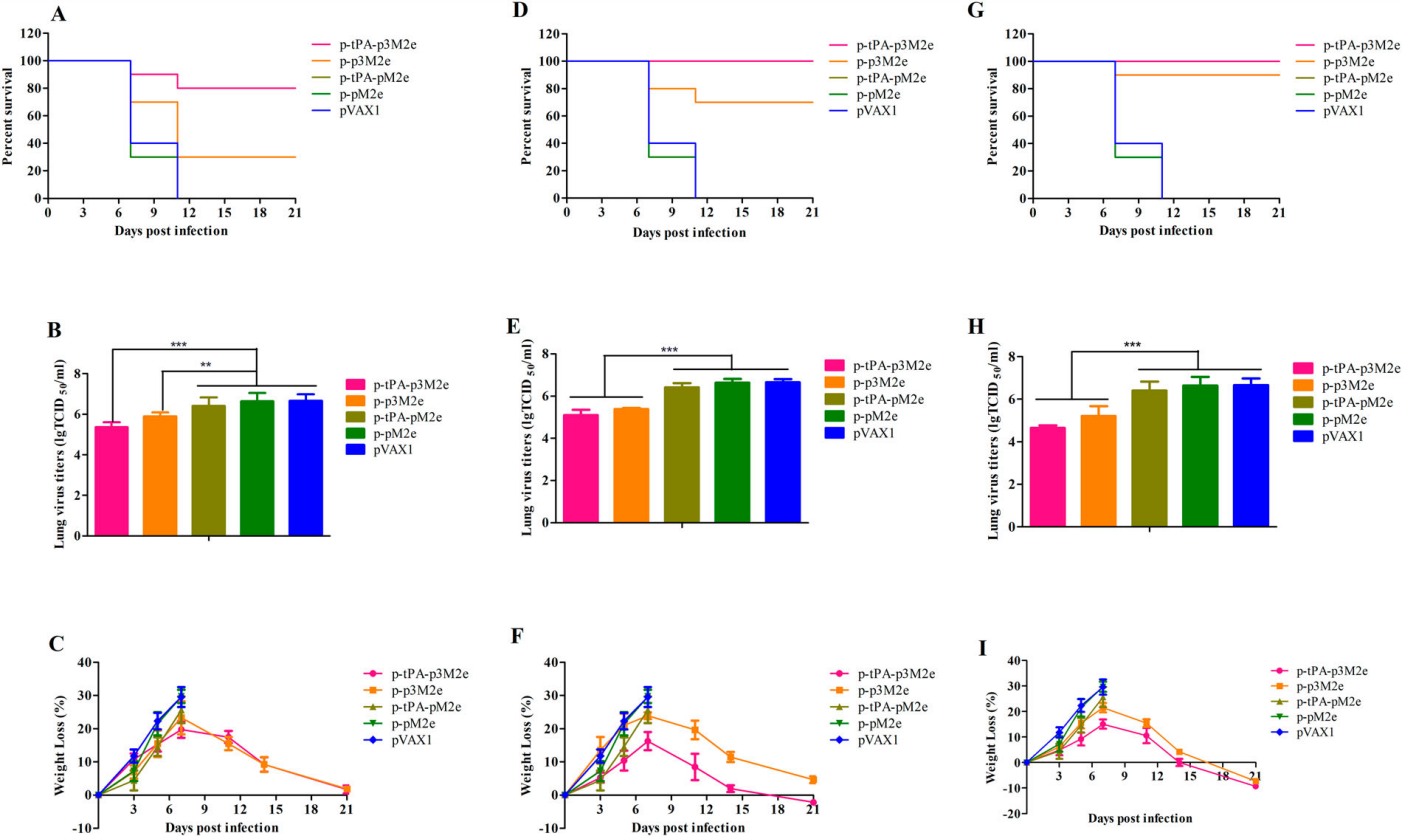
Protective efficacy against H1N1 infection. M2e DNA immunized mice were infected with a 10 × LD_50_ dose of H1N1 virus. Lungs were collected at day 3 post-infection for virus titration via TCID_50_ assay. Survival rates (A), virus titers (B) and body weight changes (C) of the immunized mice post-infection (one dose). Survival rates (D), virus titers (E) and body weight changes (F) of the immunized mice post-infection (two doses). Survival rates (G), virus titers (H) and body weight changes (I) of the immunized mice post-infection (three doses). Statistical significance was defined as ***p < 0.001, **p < 0.01.

To determine the protective effects of p-tPA-p3M2e and p-p3M2e vaccination, mice were infected with lethal mouse-adapted H1N1 virus. As shown in Figure 4(A), (D), (G), p-p3M2e and p-tPA-p3M2e DNA was immunized once, twice, or three times. In the p-p3M2e, the survival rates of mice after viral challenge were 30%, 70%, and 90%, respectively, while those for the p-tPA-p3M2e group were 80%, 100%, and 100%, respectively. Thus, the survival rate of the p-tPA-p3M2e group was better than that of the p-p3M2e group with the same number of immunizations and was particularly significant for single immunization (*p *< 0.05). In the control groups, all mice died within 11 days (Figure 4(A), (D), (G)). The above results indicated that the optimized M2e DNA vaccine significantly increased the survival rate of mice infected with homologous influenza virus.

In the lungs, mice in the control groups all had a high titer of virus, while the lung virus titer of p-tPA-p3M2e and p-p3M2e immunized mice was significantly lower than that of the control groups (*p* < 0.01). In addition, as the number of immunizations increased, lung viral titer in both DNA vaccine groups gradually decreased. Moreover, for each number of immunizations, the lung viral titer was lower in the p-tPA-p3M2e group than it was in the p-p3M2e group (Figure 4(B), (E), (H)).

We also evaluated weight loss after viral challenge. Notably, the weight loss rates of the p-tPA-p3M2e and p-p3M2e immunized groups were lower than that of the control group, but the difference was not significant (*p* > 0.05). The surviving mice in both DNA vaccination groups recovered after a slight loss of body weight, whereas mice in the control groups died within 11 days post-infection and had body weight losses of over 25% (Figure 4(C), (F), (I)).

### Optimized M2e conferred protection against heterologous avian influenza virus infection

**Figure 5.**
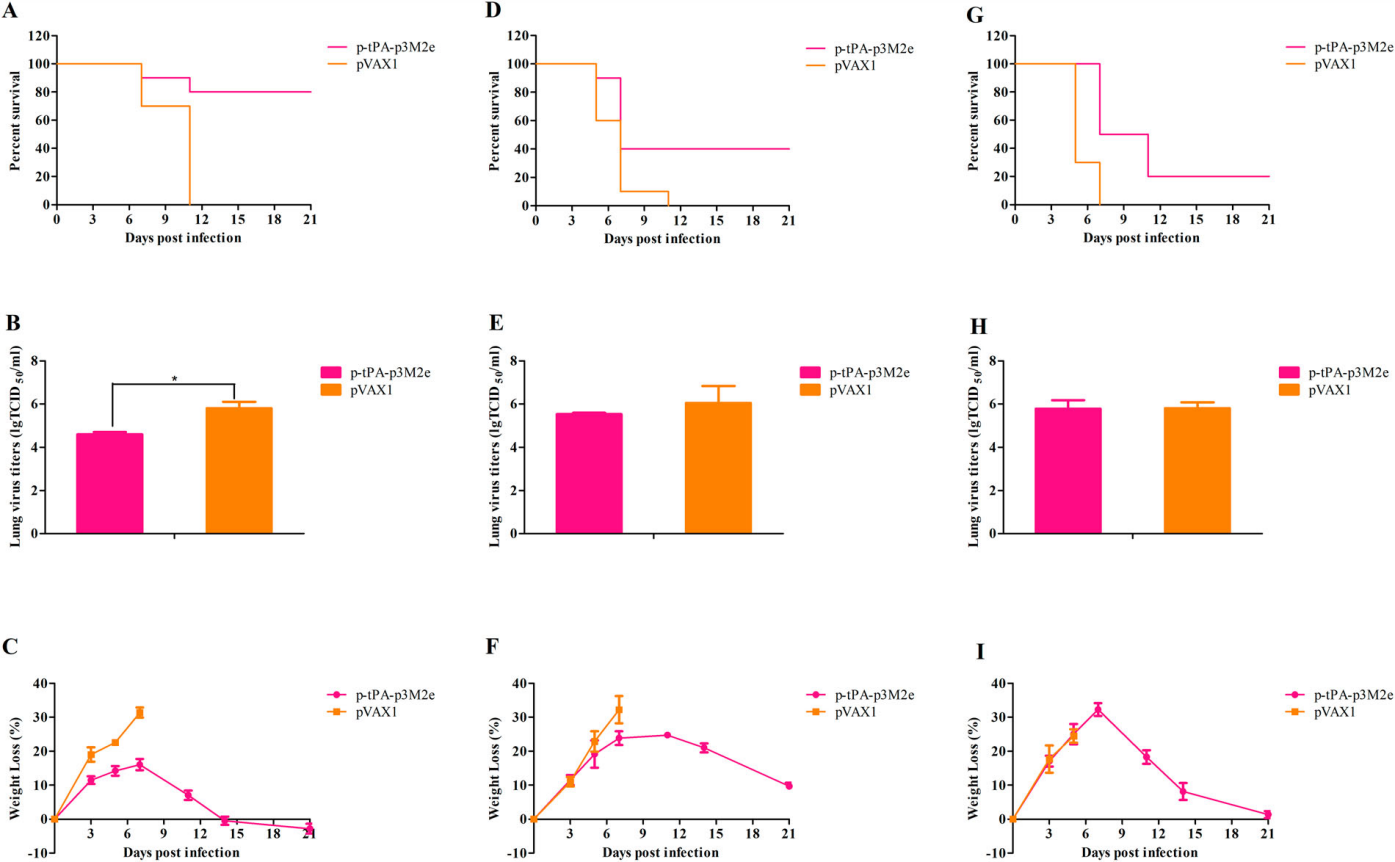
Cross-protective efficacy against the hetero-subtypic influenza viruses H9N2, H6N6, and H10N8. M2e DNA immunized mice were infected with a 10 × LD_50_ dose of each influenza virus. Lungs were collected at day 3 post-infection for virus titration via TCID_50_ assay. Survival rates (A), virus titers (B) and body weight changes (C) of immunized mice after H9N2 influenza virus infection. Survival rates (D), virus titers (E) and body weight changes (F) of immunized mice after H6N6 influenza virus infection. Survival rates (G), virus titers (H) and body weight changes (I) of immunized mice after H10N8 influenza virus infection. Statistical significance was defined as *p < 0.05.

To further determine the potential universal effects of p-tPA-p3M2e DNA vaccination against heterologous viruses, mice were infected with avian origin, mouse-adapted H9N2, H6N6, and H10N8 viruses. As shown in Figure 5(A), (D), (G), we found that the protection rates of p-tPA-p3M2e immunization against heterologous viral infection with H9N2, H6N6, and H10N8 were 80%, 40%, and 20%, respectively. However, this immunization-mediated protection was only significant for the H9N2 infected group compared to the viral challenged control group that was immunized with empty pVAX1 (*p* < 0.05). The differences between the immunized H6N6 and H10N8 groups and their respective control groups were not significant. Similarly, the lung viral titer in H9N2 challenged mice immunized with p-tPA-p3M2e was significantly lower than that in the control immunized group (*p* < 0.05), but the differences in the H6N6 and H10N8 challenged groups immunized with p-tPA-p3M2e compared to their respective control groups were not significant (Figure 5(B), (E), (H)). The weight loss rates of the p-tPA-p3M2e immunization groups were also lower than those in the respective control groups. Indeed, the surviving mice in the immunized groups began to recover by day 11 post-viral challenge (Figure 5(C), (F), (I)), while mice in the control immunized groups died within 11 days of the challenge and had body weight losses of over 25%. The above results demonstrate that vaccination with p-tPA-p3M2e protects mice from lethal infection with heterologous avian influenza strains, especially the H9N2 virus.

### Passive immunization with anti-serum from optimized M2e vaccinated mice conferred protection against lethal viral infection

**Figure 6.**
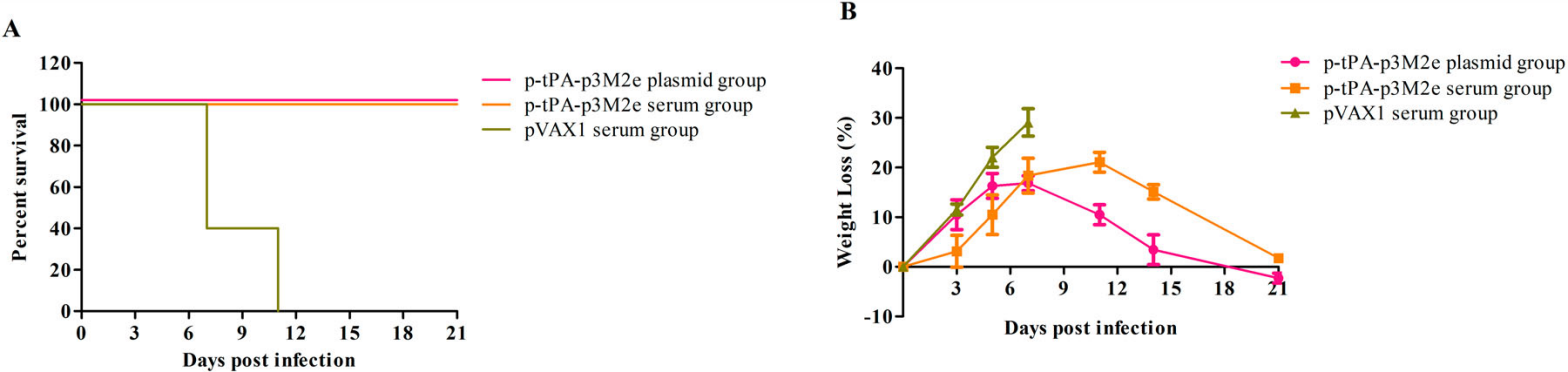
Passive protective efficacy of immune sera. Naïve mice were intraperitoneally injected with 200 µL of pooled sera collected from p-tPA-3M2e immunized mice and challenged with 10 × LD_50_ H1N1 virus 24 h after passive transfer. Survival rate (A) and body weight loss (B) were monitored after passive sera transfer.

Passive immunization was also performed to examine the protective efficacy of the immune serum of p-tPA-p3M2e or naked pVAX1 (controls) vaccinated mice. All mice injected with serum containing M2e-specific antibodies presented some clinical symptoms (eg ruffled hair and flocking together) after the challenge, but none of them died (Figure 6(A)). In contrast, mice injected with the control serum all died within 11 days of the challenge. In addition, mice injected with M2e-specific antibodies also showed less weight loss than the control groups (Figure 6(B)). The above results suggest that vaccination-mediated protection is likely provided by circulating M2e-specific antibodies.

### Protective efficacy is correlated with humoral and cellular immune responses

To better understand the relationship between protective efficiency and the humoral and cellular immune responses, correlation coefficients were calculated. With respect to the humoral immune response, the survival percentage induced by 3M2e immunization was highly related to the total M2e-specific IgG antibody levels (Figure S2A). Moreover, the survival percentage was also markedly related to the M2e-specific cellular immune response (Figure S2B). Thus, the protective efficacy of 3M2e immunization appears to be related to both the humoral and cellular immune responses.

## Discussion

At present, conventional influenza vaccines must be evaluated almost every year to follow the antigenic drift and shift of the target virus [5]. Mismatch between the circulating strains of influenza virus and vaccine strain may result in excessive influenza-related morbidity and mortality. This makes it necessary to develop a universal vaccine based on conserved epitopes, such as M2e. In this study, we created a novel M2e DNA vaccine which induced significant humoral and cellular immune responses and reduced lung virus titer and weight loss rate. This vaccine not only provided better protection against homologous viruses but also had good cross-protection effect with other heterologous viruses. To our knowledge, this is the first time an optimized M2e DNA vaccine with these particular modifications has been tested *in vivo* against both homologous and heterologous viruses.

M2e is known to have low immunogenicity. Previous studies have indicated that multiple tandem copies of M2e in a vaccine construct elicit higher M2e IgG titers than constructs containing a single copy [11–13]. In the present study, p3M2e was created and appears to induce higher production of M2e-specific antibodies than single-copy vaccines, which is consistent with previous research result. It has also been reported that secretory antigens can induce stronger immune responses than their cell-associated counterparts [16–19]. tPA is a signalling peptide located in the endoplasmic reticulum that is commonly used to promote the secretion of foreign proteins. Therefore, we coupled p3M2e with tPA to increase protein expression and secretion. Indeed, our results indicate that p-tPA-p3M2e vaccination induced higher expression and secretion of M2e protein and also induced greater humoral and cellular immune responses than p-p3M2e (Figure 3). Moreover, when challenged with homologous virus (H1N1), p-tPA-p3M2e achieved complete protection after only two immunizations, while p-p3M2e failed to achieve complete protection even after three immunizations. Taken together, these results not only demonstrate that increasing the expression of secreted M2e protein enhances its immune effects but also highlight the effectiveness of the particular modifications used in this study.

The mechanism underlying the observed M2e vaccination-mediated increase in immune protection is not completely clear at present. However, other reports have also shown that M2e-specific antibodies play an important role in protective immunity [20–22]. Indeed, anti-M2e antibody titer has been closely correlated to dose-dependent disruption of the viral life cycle and death of the infected cells via antibody dependent cell-mediated cytotoxicity (ADCC) [22,23]. In the present study, high antibody titers were induced in the p-tPA-p3M2e and p-p3M2e immunization groups, both of which demonstrated increased protection against challenge with homologous virus (H1N1). In addition, mice with M2e antibody-mediated passive immunity were also shown to be completely protected against H1N1 challenge, further indicating that M2e-specific antibodies play an important role in protective immunity. We also observed M2e-specific IFN-γ-secreting lymphocytes in the spleens of vaccinated animals, and these levels were found to be significantly correlated with protection. Notably, the p-tPA-p3M2e vaccine induced a higher T-cell response than p-p3M2e, which is likely related to the ability of the secreted protein to affect other cell types.

In cross-protection experiments on p-tPA-p3M2e DNA immunized mice, vaccination also appears to be effective against the heterologous viruses H9N2, H6N6, and H10N8. The difference in cross-protection rates (80%, 40%, and 20%, respectively) is likely due to the differences in the M2e sequence between the heterologous viruses and the H1N1 virus (Table 1). The M2e sequences of H9N2, H10N8, and H6N6 differ from that of H1N1 by four, six, and seven residues, respectively. These differences, which affect antibody binding to the heterologous M2e protein, result in failure of the vaccine to effectively clear the virus. Indeed, serum IgG antibodies from mice vaccinated with p-tPA-p3M2e appear to recognize M2e of these three viruses expressed on infected MDCK cells, but bind to them more weakly compared to H1N1-infected MDCK cells (data not shown).

**Table 1.** Sequence of matrix protein 2(M2) ectodomains.

Strain	Subtype	M2e sequence^a,b^
A/Puerto Rico/8/1934	H1N1	SLLTEVETPIRNEWGCRCNGSSD
A/Chicken/Jiangsu/7/2002	H9N2	SLLTEVETP **T**RN **G**WGCRC **SD**SSD
A/environment/Dongting Lake/Hunan/3–9/2007	H10N8	SLLTEVETP **T**RN **G**W **E**C **K**C **SD**SSD
A/Duck/Hubei/5/2010	H6N6	SLLTEVETP **T**R **SG**W **E**C **N**C **SD**SSD

^a^Variation from the PR8 M2e sequence are in boldface and underlined.

^b^Virus sequence available at www.ncbi.nih.gov/genomes/FLU.

In conclusion, we have described a potential universal influenza vaccine that provides protection against homo- and hetero-subtypic influenza in mice. Immunization with p-tPA-p3M2e, without any adjuvant, induced high level, M2e-specific antibody production, humoral/cellular immune responses, and protected BALB/c mice from lethal infections of homo- and hetero-subtypic viruses. The combined vaccine strategy offers promising prospects for further vaccine development.

## Materials and methods

### Ethics statement

All of our animal studies were carried out in strict compliance with the guidelines for the Care and Use of Laboratory Animals of the People’s Republic of China. The protocols used in this study were approved by the Committee on the Ethics of Animal Experiments of the Wuhan Institute of Virology, Chinese Academy of Sciences. All procedures were performed under pentobarbital sodium anesthesia, and all efforts were made to minimize animal suffering.

### Mice and viruses

The influenza viruses used were a mouse-adapted A/PR/8/34 (H1N1), influenza virus A/Chicken/Jiangsu/7/2002 (H9N2), influenza virus A/environment/Dongting Lake/Hunan/3–9/2007 (H10N8), and influenza virus A/Duck/HB/5/2010 (H6N6). The H9N2, H10N8, and H6N6 influenza viruses were passaged and adapted for mouse studies as described in our previous studies [24–26]. The prepared viruses were then frozen at −80°C until use. Notably, the H9N2, H10N8, and H6N6 viruses were all used in a biosafety level 2 containment facility at the Wuhan Institute of Virology, Chinese Academy of Sciences.

Specific-pathogen-free female BALB/c mice, 6–8 weeks old, were obtained from the Center for Disease Control and Prevention in Hubei Province, China. The animals were bred in the Animal Resource Center at the Wuhan Institute of Virology, Chinese Academy of Sciences. All mice were maintained in specific-pathogen-free conditions prior to infection.

### Plasmid construction and peptides

p3M2e was synthesized by GenScript Co., Ltd. Each M2e sequence was linked with a GSG4 linker (Gly–Ser–Gly–Gly–Gly–Gly). The eukaryotic expression vector pVAX1 (Invitrogen, Carlsbad, CA, USA) was used to construct the DNA vaccine. For p-p3M2e, p3M2e was digested with EcoRI and XhoI and then cloned into pVAX1 to create p-p3M2e. To construct p-tPA-p3M2e, the tPA signal sequence was ampliﬁed by PCR using the following primers: 5′-CCCAAGCTTATGGATGCAATGAAGAG AGGGCTCTGCTGTGTGCTGCTGCTG-3′ and 5′-CCGGAATTCGCTGGGCGAA ACGAAGACTGCTCCACACAGCAGCAGCACACAGCAGAG-3′. The PCR product was then digested with HindIII and EcoRI, followed by ligation into p-p3M2e to create p-tPA-p3M2e. The single-copy M2e plasmids (p-tPA-pM2e and p-pM2e) were constructed using similar methods. The plasmids were propagated in *Escherichia coli* DH5α bacteria and purified using NucleoBond® Xtra (MACHEREY-NAGEL GmbH and Co. KG).

The M2e peptide SLLTEVETPIRNEWGCRCNGSSD was synthesized by Shanghai Sangon Biological Engineering Technology and Services Co., Ltd. (>95% purity).

### Western blotting analysis

293T cells were plated onto six-well plates. Approximately 24 h after plating, the cells were transfected with the vector plasmids using Lipofectamine 2000 (Invitrogen, Carlsbad, CA, USA) according to the manufacturer’s instructions. The cells and supernatants were collected separately 48 h after transfection. The proteins in the cell lysates and supernatants were separated using non-reducing SDS-PAGE (12% gel) and blotted onto PVDF membranes. The membranes were then immunoblotted with influenza A M2 Monoclonal Antibody14C2 (Abcam, UK).

### Immunization and viral challenge

*In vivo* electroporation was performed according to the method described by Aihara and Miyazaki [27]. To evaluate the capability of DNA vaccination to protect against homologous influenza infection, a total of nine groups of 6-week-old BALB/c mice (*n* = 13 mice per group) were respectively immunized with 50 μg of p-p3M2e (three groups immunized once, twice, and three times, respectively), p-tPA-p3M2e (three groups immunized once, twice, and three times, respectively), p-tPA-pM2e (1 group immunized three times), p-pM2e (1 group immunized three times), and empty pVAX1 (1 group immunized three times). This also allowed us to examine the effects of immunization time on the protective effects of p-p3M2e and p-tPa-p3M2e. The p-tPA-pM2e, p-pM2e, and pVAX1 immunizations were used as a reference. Notably, the different plasmids (in phosphate buffered saline (PBS)) were injected into the right quadricep muscle of each mouse. After injection, a pair of electrode needles were inserted into the muscle 5 mm apart to cover the DNA injection sites, and electric pulses were delivered using an electric pulse generator (ECM830; BTX, San Diego, CA). Two weeks after the last immunization, the mice were anesthetized with pentobarbital sodium (C_11_H_17_N_2_NaO_3_) at a dose of 50 mg kg mL^−1^ for each animal and challenged with 20 μL of the viral suspension containing 10 times the LD_50_ of H1N1, the homologous virus, by intranasal drip. After viral challenge (3 days), three mice were taken from each group for blood collection, and their lungs were removed to prepare lung homogenates for measuring the virus titration. The remaining mice were observed for 21 days to record the survival rates and weight loss.

As p-tPA-p3M2e immunization induced high titer and homogenous M2e-specific antibody production, we focused on this immunization group for the heterologous virus challenge experiment. A total of 78 mice were divided into six groups (*n* = 13 mice per group). Three groups were immunized three times with 50 μg of p-tPA-p3M2e at 2-week intervals. The other three groups were immunized with empty pVAX1 as a control. Two weeks after the third immunization, the p-tPA-p3M2e and pVAX1 immunization groups were challenged with nasal drips of 10 times the LD_50_ of H9N2, H10N8, and H6N6 influenza viruses, respectively. The mice were observed for 21 days after viral challenge, during which weight loss and survival rate were recorded. Virus titers in the lungs collected at day 3 post-challenge were tested via TCID_50_ assay.

### Specimen collection

Blood was collected 14 days after each immunization, and serum was isolated for antibody detection. Serum samples were stored at −20°C until use. Mice from each group (*n* = 3) were sacrificed 3 days after viral challenge with H1N1, H9N2, H10N8, or H6N6 and their lungs were isolated. Lung homogenates were centrifuged at 1000 rpm for 10 min. After centrifugation, the supernatants were collected for viral titration via TCID_50_ assay.

### Enzyme-linked immunosorbent assay

M2e-specific antibodies were tested using plates coated with 10 μg/mL of M2e synthetic peptides in 96 well plates by ELISA as described previously [28]. To identify the M2e-specific antibody isotypes, the serum samples were diluted in 1% bovine serum albumin-PBS and incubated for 2 h at room temperature (RT). After washing, 100 μL of biotinylated anti-mouse IgG, IgG1, and IgG2a (Southern Biotechnology Associates, Inc., USA) conjugated to streptavidin-horseradish peroxidase (HRP) were added and incubated for 1 h at RT. The samples were then incubated with 3,3′,5,5′-Tetramethylbenzidine (TMB) substrate for 10 min, and then the reaction was stopped with 1 M phosphoric acid. The end-point ELISA titers are expressed as the highest dilution that yielded an optical density greater than the mean plus two times the standard deviation of a similarly diluted negative control sample.

### Whole-cell ELISA

Whole-cell ELISA for M2e-specific antibodies was performed in 96 well plates using a previously published procedure with some modifications [29]. In brief, Madin-Darby Canine Kidney (MDCK) cells were grown in 96-well culture plates in minimum essential medium (MEM) complete medium containing 10% fetal bovine serum (FBS) at 37°C until the cells were almost conﬂuent. The cells were then incubated with 10^6^ EID_50_ of H1N1 viruses (100 μL) in PBS or with medium alone (for uninfected controls). After incubating for 2 h at 37°C, 200 μL of complete medium was added to each well and plates were incubated at 37°C for 16 h. The plates were then washed with PBS and ﬁxed with 10% formalin at RT for 10 min. Then, the cells were washed three times with PBS and blocked with 200 μL/well PBS/3% BSA for 2 h at 37°C. Serial dilutions of tested sera were added to the wells and incubated for 1–2 h at 37°C. After incubation, the cells were washed and incubated with HRP-conjugated goat anti-mouse IgG (Boster, Wuhan, China) for 1 h at 37°C, followed by TMB Horseradish Peroxidase Color Development Solution for ELISA (Beyotine Biotechnology, Shanghai, China) for 10 min at 37°C. The reaction was stopped with the addition of 50 μL of 2 mol/L H_2_SO_4_ and the OD_450_ was measured on a microplate spectrophotometer. Data reﬂect the mean change in OD (infected–uninfected) of triplicate wells per sample.

### Viral titration

MDCK cells were seeded (*n* = 4) at 2 × 10^4^ cells per well in a 96-well plate. After being cultured for 12 h, the cells were infected with 100 μL of a 1/10-dilution series of lung homogenate supernatant and incubated at 37°C for 1 h. The supernatants were then replaced with 200 μL of serum-free Dulbecco’s modified Eagle medium (DMEM) containing a cocktail of antibiotics. The cytopathic effects in the infected MDCK cells were observed daily. After incubating for 3 days, virus titer was assayed in the supernatant by measuring hemagglutinating activity and processing these data using the calculations of Reed and Muench.

### Enzyme-linked immunospot (ELISPOT) assay

Splenocytes were isolated from mice for ELISPOT assays 2 weeks after the final immunization. According to manufacturer’s instructions (U-CyTech, Netherlands), the immunospot plates (Millipore, Bedford, MA) were coated with rat anti-mouse interferon (IFN)-γ monoclonal antibody and incubated at 4°C overnight. The plates were washed three times with sterile PBS and then blocked with 200 µL of blocking solution R. Next, 2 × 10^5^ splenocytes were added to the wells in triplicate. After stimulating the splenocytes with 10 µg/mL of M2e peptide at 37°C for 18 h, biotinylated anti-mouse IFN-γ antibody was added to each well and incubated at 37°C for 1 h. Subsequently, diluted streptavidin-HRP conjugate solution was added and incubated at RT for 2 h. Finally, the plates were treated with 100 µL of AEC substrate solution and incubated at RT for 20 min in the dark. The reaction was stopped by washing with demineralized water. Spots were quantified with an ELISPOT reader (Bioreader 4000; Bio-sys, Germany).

### Passive immunization and viral challenge

To prepare a high titer of M2e-immune serum, BALB/c mice (*n* = 30) were immunized with 50 μg p-tPA-p3M2e vaccine construct as described above. A second group of mice (*n* = 30) was immunized with empty pVAX1 vector. Mice were immunized three times at 2-week intervals. Two weeks after the final immunization, 20 mice were killed and the other 10 mice from each group were used as controls. Blood samples were collected, and serum was prepared by clotting the blood at 37°C for 30 min followed by centrifugation. A total of 10 naive mice were immunized by tail vein injection with 300 μL of pooled serum from the mice immunized with p-tPA-p3M2e. Another group of 10 naive mice received the same amount of pooled serum from the mice immunized with only pVAX1. After 24 h, the mice were anesthetized and challenged with H1N1 influenza virus as described above. Mortality and morbidity (weight loss) were monitored for 21 days after challenge [30].

### Statistics

Statistical analyses were conducted by one-way analysis of variance with Bonferroni post-test using SPSS for Windows software (ver. 17, SPSS Inc., Chicago, IL, USA). An unpaired two-tailed Student’s *t-test* was used to compare the means between different groups. A *p*-value less than 0.05 was considered statistically significant. The differences in survival rate between the groups were analysed using the Kaplan–Meier method. Results are expressed as the means ± standard deviations (SD).
